# Editorial: Brain hemispheric specialization and its pathological change revealed by neuroimaging and neuropsychology

**DOI:** 10.3389/fneur.2022.1071148

**Published:** 2022-11-04

**Authors:** Alessia Sarica, Andrea Quattrone, Margit Jehna, Maria Grazia Vaccaro

**Affiliations:** ^1^Department of Medical and Surgical Sciences, Neuroscience Research Center, Magna Graecia University of Catanzaro, Catanzaro, Italy; ^2^Department of Radiology, Division of Neuroradiology, Vascular and Interventional Radiology, Medical University of Graz, Graz, Austria

**Keywords:** brain asymmetry, brain lateralization, neuroimaging, neuropsychology, brain hemispheric differences

Hemispheric specialization of the human brain is a characteristic feature of functional cortical organization (1). Morphological, functional, metabolic and connectivity asymmetries of brain regions were traditionally correlated to optimal information processing, language function, visuospatial task, attention, and many aspects of emotion (2).

Brain lateralization varies with the normal aging (3), but more interestingly, brain asymmetry could increase or decrease in presence of a neurological or neurodegenerative disease (2, 4). Thus, peculiar deviations from normal asymmetry of one or more brain regions may serve as a neuroanatomical marker (2) or as a risk factor (5). In other words, the existence of asymmetry in brain regions where the symmetry is expected or, on the contrary, the absence of asymmetry where asymmetry is expected could be often indicative of a psychiatric disorder, a neurological or neurodegenerative disease (2).

The aim of the present Research Topic was to collect scientific works that explore brain hemispheric lateralization and asymmetry through Neuroimaging and Neuropsychology, as well as to bring light to their relationship with behavioral, cognitive, and symptomatic alterations in neurological and neurodegenerative diseases. A total of five articles were collected here: one was focused on the normal functioning of the human brain in language production and visuospatial attention (Jia et al.), two works explored the pathological asymmetry in Depressive disorders (Sun et al.; Zheng et al.), one article investigated brain changes in patients with stroke (Park et al.) and another one studied patients with Crohn's disease and psychological disorders (Huang et al.).

The work by Jia et al. focuses on the well-known lateralized functions of language production (left hemisphere) and visuospatial attention (right hemisphere), and specifically on their relationship in a cohort of right-handed healthy students. The authors acquired structural Magnetic Resonance Imaging (MRI) and functional near-infrared spectroscopy (firs) data of the participants to measure the cerebral lateralization while performing the picture-naming and the landmark tasks. In particular, the degree of hemispheric lateralization was quantified according to the fNIRS activation difference between the left and right hemispheres and by the lateralization index. The findings showed that the left hemisphere (inferior frontal gyrus) was the predominant activated side by the picture-naming task, while the right hemisphere (superior parietal lobule) was activated by the landmark task. Moreover, the absence of correlation between the laterality indices of these two tasks corroborated the hypothesis that different cognitive tasks may produce lateralized processing which are independent of each other (Jia et al.).

Two of the collected works investigated major depressive disorders (MDD), demonstrating an impairment of inter-hemispheric functional connectivity (FC) through resting-state functional MRI (rs-fMRI) analyses (Sun et al.; Zheng et al.). More in detail, Zheng et al. found that MDD patients had a decrease of the homotopic FC in the bilateral medial prefrontal cortex. Together with FC analysis, Zheng et al. found through Diffusion Tensor Imaging (DTI) that MDD patients had lower fractional anisotropy (FA) (6) of the anterior corpus callosum (CC) compared with control subjects. The FC alteration of bilateral medial prefrontal cortex and the lower FA of the anterior CC were correlated to each other and associated with depression severity in MDD patients. Similarly, Sun et al. found a significant correlation between FC disconnection of peculiar, lateralized brain regions and the depression severity scale in first depressive episode patients (left dorsolateral prefrontal cortices and right precuneus) and recurrent depressive episode patients (left posterior cingulated cortices and right inferior temporal gyrus). Interestingly, both articles (Sun et al.; Zheng et al.) demonstrated that interhemispheric FC were associated with clinical severity in patients with depressive disorders.

Park et al. investigated the lateralized injury of the Parieto-Insular Vestibular Cortex (PIVC) by applying DTI analysis and explored the association between the PIVC projection pathways and the gait impairment assessed with the motricity index (MI) and the functional ambulatory category (FAC) scale. Probabilistic tractography was used for reconstructing the projection pathway to the PIVC and the diffusion metrics of both affected and unaffected side were extracted and compared. The correlation between diffusion metrics and motor function scores revealed a mild positive correlation between fractional anisotropy (FA) and motor function in the affected PIVC, while in the unaffected hemisphere, PIVC FA correlated negatively with MI. Importantly, the authors investigated the association of DTI parameters of the affected PIVC with the changes in the gait and motor function recovery process after rehabilitation. These correlations suggested that neuroplasticity may have compensated the affected PIVC by inducing the hyperactivation of the contralateral unaffected side without restoration of motor function (Park et al.).

In the fifth collected article, Huang et al. studied patients affected by Crohn's disease (CD) with psychological disorders, by analyzing brain regional homogeneity (ReHo) data extracted from rs-fMRI. The CD patients had widespread cerebral alterations compared to healthy controls, and these alterations were correlated with obsessive-compulsive scores and depression scale. These results demonstrated that CD patients could present both clinical intestinal symptoms and psychological disorders, which can affect each other (Huang et al.).

In conclusion, this Research Topic highlighted the importance to investigate the brain hemispheric lateralization in the normal functioning (Jia et al.), in depression disorders, (Sun et al.; Zheng et al.), in stroke (Park et al.) and in CD (Huang et al.). Moreover, we demonstrated that regional deviations from normal asymmetry are often correlated with clinical, motor and psychological scores, thus providing a valuable support in the clinical evaluation, diagnosis and prognosis of patients.

## Author contributions

AS produced the first draft. AQ, MJ, and MGV revised the draft. All authors contributed to the article and approved the submitted version.

## Conflict of interest

The authors declare that the research was conducted in the absence of any commercial or financial relationships that could be construed as a potential conflict of interest.

## Publisher's note

All claims expressed in this article are solely those of the authors and do not necessarily represent those of their affiliated organizations, or those of the publisher, the editors and the reviewers. Any product that may be evaluated in this article, or claim that may be made by its manufacturer, is not guaranteed or endorsed by the publisher.
